# Palliative medicine physicians’ experiences using the Numeric Rating Scale for pain assessment in patients with advanced cancer: a qualitative study

**DOI:** 10.1136/bmjopen-2025-102830

**Published:** 2026-01-06

**Authors:** Lisa Martinsson, Margareta Brännström, Per Fransson, Sofia Andersson

**Affiliations:** 1 Diagnostics and Intervention, Oncology, Umeå University, Umeå, Sweden; 2 Clinical Sciences, Professional Development, Umeå University, Umeå, Sweden; 3 Nursing, Umeå Universitet, Skellefteå, Sweden; 4 Nursing, Umeå Universitet, Umeå, Sweden; 5 Regional Cancer Centre North, Norrlands universitetssjukhus, Umeå, Sweden

**Keywords:** PALLIATIVE CARE, PAIN MANAGEMENT, Cancer pain

## Abstract

**Abstract:**

**Objectives:**

This study aimed to describe palliative medicine physicians’ experiences performing pain assessment using the Numeric Rating Scale (NRS)—one of the most widely used pain assessment tools—for patients with cancer receiving specialised palliative care.

**Study design:**

This qualitative study used reflexive thematic analysis.

**Setting:**

The study was conducted in specialised palliative care settings.

**Participants:**

Semi-structured interviews were conducted with 14 palliative medicine physicians in specialised palliative care.

**Analysis:**

The interviews were transcribed and analysed using reflexive thematic analysis.

**Results:**

Four themes were identified: ‘Striving to create a shared understanding’, ‘Meeting individual needs’, ‘Interpreting and managing ratings’ and ‘Importance of organizational structures’. This can be seen as a process that moves from creating a shared foundation through individual patient meetings and handling NRS ratings to organisational-level challenges.

**Conclusions:**

The study shows the complexity needed within palliative cancer care when using the most common pain assessment tool in Sweden, the NRS. The tool may seem simplistic, but, as shown in this study, the physicians found interpreting the assessments challenging for the whole team. This complexity should be incorporated into future healthcare education and training within the palliative care area, where patients often have chronic pain conditions in combination with cognitive impairment. Future research needs to focus on developing reliable pain assessment methods for patients who are cognitively impaired because of the cancer.

**STRENGTHS AND LIMITATIONS OF THIS STUDY**

- A strength in this study is that interview participants were recruited from various geographical settings in Sweden, which is important because local traditions differ throughout the country.
- A strength is that physicians from both inpatient care and ambulatory home care were interviewed to get a representative view from both settings.
- The interviewed physicians in this study were all specialists, and almost all were specialists in palliative medicine.
- This context is palliative medicine in Sweden, and the findings might be influenced by culture and other aspects regarding the context, and therefore not directly transferable to other settings.

## Introduction

 Pain is common among patients with cancer, and symptom management, including pain alleviation, is central in their palliative care.^1^ Pain experienced by patients with advanced cancer is dynamic, complex and non-linear in its nature,^2^ thus making it difficult to measure and characterise fully. Despite that, standardised pain assessment focusing on pain intensity is important and well-established for pain management in clinical practice. The European Society for Medical Oncology guidelines state that initial and ongoing assessment of pain should be an integral part of cancer care.^3^ Similar recommendations exist in Swedish guidelines.^4^

The most common scales used for pain intensity assessment according to the literature are the Numeric Rating Scale (NRS) and the Visual Analogue Scale (VAS). NRS is based on a scale from 0 to 10, where 0 is no pain and 10 is the worst possible pain, where the patient enters a number on the scale. VAS is a non-numbered scale but is very similar to NRS.^5 6^ Our research group has conducted a nationwide study about which pain assessment instruments are used in Sweden during the last week of life for patients with cancer within specialised palliative care and found that the NRS was most commonly used, followed by the VAS (unpublished data).

To be able to use NRS and VAS, the patient must understand and answer the question, which is a limitation during severe illnesses because many patients are tired and cognitively affected. It has been previously described that NRS is preferred as a pain assessment method by patients over a variant called VAS qualitative. This scale asks patients their pain level along a continuum of ‘Good Day’, ‘Average Day’ or ‘Bad Day’.^7^ Using a tool that needs cognitively intact patients in a context where the patients often are cognitively affected seems to require careful attention from the healthcare team, but healthcare professionals’ point of view of using the NRS in this context was not found in the literature.

The NRS can be used for one aspect of pain, but many other aspects can be interesting in the patient–physician interaction to promote good pain management. Willems *et al* suggest that an addition to the NRS in the form of the (non) acceptable pain evaluation is used.^8^ In a study by Erol *et al* patients with advanced cancer and cancer pain reported that they experienced that nurses did not perform systematic and proper pain assessments.^9^

Although the NRS is the most common tool used in the palliative care context in Sweden as well as a common tool described in international literature, no qualitative description of patients’ ‘experience of using NRS, nor any study describing healthcare professionals’ experience of using NRS as a pain assessment tool for patients with cancer has been found in the literature.

### Aim

This study aimed to describe palliative medicine physicians’ experiences performing pain assessment using the NRS for patients with cancer receiving specialised palliative care.

## Methods

This is a reflexive thematic analysis study based on semi-structured interviews.

### Study participant recruitment

Inclusion criteria were 18 years or older, consent to the study and a physician working in specialised palliative medicine. Exclusion criteria were language barriers hindering an interview in Swedish or being unable to perform a digital interview.

Purposeful sampling was used to achieve variation regarding sex, age and clinical background. Study participants with much and little experience were recruited to reflect everyday clinical practice. Managers within specialised palliative care were contacted to obtain permission for physicians to be interviewed during work hours and to mediate contact with the employed physicians.

Persons suitable as study participants were asked about participation both orally and in writing. In the event of a preliminary positive answer, an interview was booked. The oral information was repeated at the meeting before the interview began, and written consent was obtained. This procedure resulted in interviewing 14 people. Out of these, 13 were palliative medicine specialists. To become a palliative medicine specialist in Sweden, another first specialist training is mandatory. Specialists who proceed to palliative medicine training are often specialists in oncology, family medicine or geriatrics. Background information about these is shown in table 1. No person who was booked for an interview declined participation and no person dropped out of the study.

**Table 1:** Background information about the interviewees (n=14)

Palliative medicine specialist	Yes	13
	No	1
Primarily working with	Ambulatory home care	5
	Inpatient care	1
	Both ambulatory home care and inpatient care	8
Age (years)	Median	50
	Mean	51
	Range	39–68
Sex	Women	9
	Men	5

### Interviews

A semi-structured interview guide was used (see online supplemental material). Four interviews were conducted face-to-face at the person’s workplace and 10 were conducted digitally. Before each interview, the interviewer introduced themselves, described their background and explained the purpose of the study. The first and the last authors served as interviewers. The first author is a female physician working in oncology and the last author is a female registered nurse and process leader within palliative care. Both have prior research experience conducting study interviews and in qualitative research.

The interviews lasted 9–49 min each, with a median of 29 min and a mean of 27 min. They were audio recorded and later transcribed verbatim. Only the interviewed person and the researcher(s) were present during the interviews. Memos were written when needed to record practical issues, such as technical problems, that arose during the interview process. Transcripts were not returned to participants for comments or corrections, and participants were not asked to provide feedback on the findings.

### Data analysis

The research group considered the data material rich after 14 interviews, and no further data collection was deemed necessary. A reflexive thematic analysis, following the six-phase approach outlined by Braun and Clarke,^10 11^ was conducted. This method was chosen for its flexibility and its emphasis on the researcher’s active role in identifying and interpreting patterns of meaning across the data set.

First, familiarity with the data was established through repeated readings of the transcripts by all authors. Initial observations and reflections were noted to guide the subsequent coding. Next, the data were systematically coded using an inductive approach. Coding was conducted using Microsoft Word to allow close engagement with the data. The codes were then reviewed and organised into initial themes based on shared meaning and conceptual similarities through ongoing reflection and discussion within the research team. The initial themes were refined by comparing them with the coded data and the full data set. The results were continuously discussed among the authors, and four themes were agreed. The themes were revised to clarify their content until consensus was reached. To enhance rigour and trustworthiness, an effort has been made to describe the analysis procedure and give readers the opportunity of following the interpretation and examples from original text are provided (table 2).

**Table 2:** Meaning units, codes, themes

Meaning units	Codes	Themes
Furthermore, I often tell the patients ‘If you imagine a thermometer from 0 to 10, where 10 is the worst imaginable, and 0 is no problem, how is it right now?’ ‘Can you put a number on how much trouble you feel?’ Interview 10	Using a thermometer as a metaphor	Striving to create a shared understanding
‘Then there is a group of patients who find it difficult to express. They may not have this cognitive ability or are too weak or tired. So, then you have to find other strategies to find out how they feel in order to relieve pain’. Interview 2	Find strategies for patients who find it difficult	Meeting individual needs
And then it’s my task to help my patient to reflect a bit. Is it pain in a leg, or is it pain in the heart—that is, mental pain. And of course, you can have both, and one affects the other. Interview 3	To support the patient in exploring their experience of pain, encompassing both physical and psychological dimensions	Interpreting and managing ratings
‘It is unclear how we should document things, and that means we lack the structure. We can’t find it. We want to, but we haven’t quite found the map yet’. Interview 8	Lack of structures	Importance of organisational structures

### Patient and public involvement

There was no involvement from patients or the public in planning this study.

## Results

Four themes were identified: ‘Striving to create a shared understanding’, ‘Meeting individual needs’, ‘Interpreting and managing ratings’ and ‘Importance of organizational structures’. These form a process from establishing shared ground, to the individual patient encounter, to handling the NRS rating and finally to the organisational-level challenges.

### Striving to create a shared understanding

The NRS was described as a useful tool for communication between physicians and patients to create a shared understanding. From the physician’s perspective, it is easy to use and requires no preparation or printed instructions. They described it as a way to begin addressing pain and pain management, while acknowledging that it does capture all important aspects of a comprehensive pain analysis. It was frequently used during the first appointment or home visit, for example, when admitting the patient to specialised palliative home care.

The physicians reported that patients usually learn the scale and how to use it. It may not be easy at first, but over time many patients spontaneously report their pain using the NRS without prompting. Physicians found it helpful for pain management when patients learnt to report pain with NRS. Many clinicians use strategies for patients who find it difficult, such as showing the VAS or describing the NRS as a thermometer. It often takes time for patients to learn.

Some patients need encouragement to report their pain. Some use different words, sometimes due to dialect, and do not describe their discomfort as ‘pain’. Others perceive cancer pain as natural and expected and therefore do not report it spontaneously. It helps when the physician explains that pain reporting is important to healthcare. One strategy was to draw a parallel to measuring blood pressure when starting and evaluating antihypertensive treatment.

I like to use the examples of blood sugar and blood pressure, and no one is surprised if you have to check something sometimes. (Interview 3)

The NRS questions and instructions on how to use the scale varied between physicians. Some provided examples of worst thinkable pain, such as labour pain or fractures, and mild pain, such as mosquito bites, while other physicians avoided examples to reduce the risk of leading the patient. One approach used was to relate the scale to a previous painful incident.

Then I usually say ‘if you were to chop off a leg, and you are conscious, not sedated or something’ just to get an understanding that 10 is really the worst they can think of. (Interview 2)

Some patients initially misunderstand the NRS and think that 0 is the worst possible pain and 10 is no pain. This misunderstanding is usually easy to correct when the physician explains the scale. Some patients rate a number higher than 10, for example, 14; this is interpreted as expressing unbearable pain and is another way of indicating 10.

### Meeting individual needs

The NRS is widely used and, in most cases, a helpful scale for individual pain assessment and management. However, there are situations in which physicians modify their use of the NRS because of patient factors. Physicians tend to avoid the NRS in late palliative phases because they do not want to disturb patients who are severely ill; this is seen as a matter of dignity and sparing patients unnecessary harm. Instead, observatory assessment tools or dichotomic questions, such as ‘Are you in pain?’, are used.

I don’t ask the question if they are really tired. (Interview 1)

Most cognitively intact patients can reliably use the NRS scale. However, a few physically active patients cannot use the NRS or may always answer with the same number, so physicians must find other ways to assess pain. Factors the physicians associated with difficulties in answering the NRS mentioned in the interviews were cognitive impairment, male sex, older age, low educational level, different cultural background, a personality to which the scale simply does not fit and lack of previous experience of severe pain. Some physicians speculated that this could be related to problematic childhood attachment or inherent difficulties in interpreting and communicating their internal experiences. Others most often interpreted the difficulties of using the NRS as a cognitive impairment. Some had not reflected on why the patient could not answer the NRS and quickly adopted other measures to communicate about pain with the patients. Other participants argued that many patients with cognitive impairment could use the NRS if the scale is explained correctly and the patient is asked to focus on their pain at the moment, whereas fewer patients with cognitive impairment can describe the pain experienced previously. Additional factors hindering use of the NRS are speech impairment, hearing impairment and lack of a common language.

Then it was probably patients over 70. Rarely the younger ones. Or, the younger ones justify it. They say this is not enough because I can’t find myself. That is not enough to describe my situation. The older ones just say ‘Uh, go away’. (Interview 3)

Patients sometimes express displeasure about rating their pain even though they are able to do so. The physicians felt this was often because the patients felt pressured to answer. It can be difficult for patients to respond because of the hindrances described above, such as mild cognitive impairment, which can be frustrating. Frustration can also be caused when the healthcare staff do not use the patients’ answers. For example, patients who do not want analgesics are sometimes reluctant to describe their pain as a number if they believe the rating will be used only for documentation. Physicians also felt like the NRS did not fit all patients. Pain is sometimes so multidimensional that the NRS scale is too narrow for both patients and physicians.

### Interpreting and managing ratings

The NRS ratings were described as blunt and not always easy to interpret. The pain experience is complex and not fully explained by a number. The physicians emphasised their role in supporting the patient in exploring their experience of pain, encompassing both physical and psychological dimensions. Sometimes, patients rate different pain levels depending on the profession of the healthcare staff. Previously, there was a practice that anyone who rated 3 or higher should receive pain relief, but this is now questioned and is no longer clinical practice. A common way to qualify the rating is to ask whether the patient wants pain relief and whether their pain level is acceptable or whether they wish to lower it.

The physicians also often looked for signs of severe acute pain and asked the patient again if they appeared to be in pain despite a low rating. They also sought to qualify a high rating when a patient appeared unaffected by pain. The physicians reported that the patients sometimes did not understand the scale even when giving a clear answer. In cases where a patient rated 9 or 10 but the physician did not believe the patient was in severe pain, physicians sometimes documented the number with a comment about the patient not appearing to be in severe pain, or they omitted the NRS rating from the visit record.

Sometimes I can probably write the [high] number but maybe with a clarification ‘but can still participate in the conversation’, despite the high number. But sometimes it can also be that I choose not to write it down at all. (Interview 1)

Some unexpected ratings can be difficult for the team to handle. This can occur when a patient rates very high on the NRS despite several measures being taken, such as administering high doses of opioids. The team may describe this as the patient not rating correctly or believe that the patient cannot be in such pain. These situations are challenging to manage. Several physicians reported that such cases often reflect multifactorial pain, including anxiety and existential distress, sometimes described within the total pain concept. They expressed their role as trying to find the cause of the pain, which in some cases may not respond well to opioids. Nurses and nursing assistants are regarded as important sources of information for this assessment, but they emphasise that the patient’s experience is the most important.

And then I think that there I have an assignment as a physician to sort of put myself on the patient’s side vis-a-vis the team and say ‘We have to assume that the patient is the one who knows how it feels’. (Interview 10)

### Importance of organisational structures

Several physicians emphasised the need for better routines and structures for pain assessment and management. While the NRS is used to assess pain levels during initial home care visits, it is not consistently used in subsequent visits. Often, physicians do not have enough time to evaluate the effectiveness of pain medication because they typically administer opioids during the visit and cannot stay long enough for a thorough reassessment. Additionally, pain is sometimes assessed before but not after opioid administration, making it challenging to gauge the medication’s impact. Physicians also noted a need for established routines for team-based follow-up after assessing pain.

The absence of NRS ratings in medical records becomes problematic when palliative medicine is consulted for patients in hospital wards. Even when patients report pain, there is often no documented pain assessment. The effectiveness of routines and structures for NRS assessments varies widely across clinics and hospitals.

There is a concern that patients may experience harm or frustration from NRS assessments if they are conducted without proper structures and follow-up procedures in place. The interviewed physicians emphasised the importance of tailoring pain-assessment tools to each patient’s unique needs. Sometimes, organisational decisions have mandated NRS assessments, leading to confusion among healthcare staff and annoyance for patients. Furthermore, there needs to be greater consistency in how and where NRS ratings are documented.

Unfortunately, it’s not always me who evaluates afterwards, which many times would certainly have been good, to meet them myself. (Interview 1)

## Discussion

This study examining physicians’ experiences of using the NRS in palliative oncology care showed that the NRS is a tool for patients and physicians to facilitate pain communication, but that the physician’s specific role in the team is to filter the patients’ ratings through their global assessment to make them useful for analgesic decisions. Although it is a valuable tool, individual considerations are most important when asking for NRS ratings, and the tool is not suitable for every patient or situation. If used when not suitable, it could potentially disrupt communication. For the NRS to support better pain management, there is a great need for organisational structures. This highlights the importance of assessing the NRS several times during the disease course and making appropriate long-term decisions. To our knowledge, no other qualitative study has been published describing physicians’ experiences with the NRS overall or in this palliative oncology care context.

In a study by van Dijk *et al*, patients reported that their NRS ratings postsurgery were influenced not only by their pain level but also by how they felt the number would be perceived and whether they wanted pain medication.^12^ These findings correspond with our results: the patient’s rating sometimes needs to be nuanced by a physician to give the healthcare providers the best picture of the pain experience. They also reflect that asking whether the patient wants analgesics may be more important than the exact NRS number.

Eriksson *et al*^13^ also examined patients’ perspectives on postoperative pain ratings using the NRS. Like our findings, they describe the NRS as a communication facilitator, which also places demand on clinical routines. This suggests that, despite different contexts (acute vs more chronic pain), pain assessment presents the same type of challenges for healthcare services. The strategy of discussing whether the patients needed pain medication when they could not answer the NRS has been described as the (non)acceptable pain evaluation scale.^8^ In that study, oncology patients who indicated non-acceptable pain had a mean NRS of 6.5 while patients indicating acceptable pain had a mean NRS of 1.6.^8^

Bergh *et al*^14^ examined how patients with cancer interpret and respond to the Edmonton Symptom Assessment System, which is a palliative care assortment tool that includes two pain items. They found that patients struggled with the wording ‘worst possible’ pain, because it made them feel and think about negative things. This is a conceivable drawback to the NRS ratings in a palliative context, where physicians might also be hesitant to ask patients to rate very high levels of.

We found that there are situations where the NRS is not suitable. As previously shown in the literature, cancer pain is complex^2^ and not fully described using a pain-intensity scale. For patients with trouble comprehending the 11-point scale, a simpler approach (eg, asking whether the pain is mild, moderate or severe) can be tried. Our results also suggest that asking whether the patient wants pain medication (yes or no) can be sufficient in many cases. However, there are situations where a verbal approach is not possible, for example, when the patient is in the late palliative phase and is non-responding due to extreme fatigue. In such cases, observation pain assessment could be of value, but the most commonly used one in Sweden, the Abbey Pain Scale, has been shown to not be a sufficiently valid and reliable tool in this context.^15^ Future research needs to focus on developing pain assessment methods for patients who cannot self-report pain. Future studies can also focus on the view of the patients and family members regarding pain assessment in palliative oncology care.

### Methodological considerations

This qualitative study examined healthcare professionals’ experiences of the NRS as a pain tool in Sweden. A strength of this study was that the participants came from different geographical settings and local traditions. To further increase representation of different local views on pain assessment, physicians from both inpatient care and ambulatory home care were interviewed. The interviewees were all specialists in their primary specialty, and most of them were also palliative medicine specialists. All interviewees were physicians, so the views of nurses or other healthcare professionals were not examined. These findings are specific to palliative medicine in Sweden; they may be influenced by cultural and contextual factors and therefore not directly transferable to other settings.

## Conclusions

The study shows the complexity needed within palliative cancer care when using the most common pain assessment tool in Sweden, the NRS. The tool may seem simplistic, but as shown in this study, the physicians experience interpreting the assessments as challenging for the whole team. Together, the themes found in this study underscore the complexity of integrating patient-centred care with systematic evaluation and institutional frameworks. This complexity should be incorporated into future healthcare education and training within the palliative care area, where patients often have chronic pain conditions in combination with cognitive impairment. Training and support for healthcare teams are needed to ensure that NRS assessments are meaningful, consistently documented and followed appropriately. Clear organisational routines, interdisciplinary collaboration and flexibility in using the NRS pain assessment tool are essential for achieving truly person-centred palliative care. Future research needs to focus on developing reliable pain assessment methods for patients who are cognitively impaired because of cancer and cannot self-report pain.

## Supplementary material

10.1136/bmjopen-2025-102830 online supplemental file 1

## Data Availability

No data are available.
