# Supplementary material for: Palliative medicine physicians’ experiences using the Numeric Rating Scale for pain assessment in patients with advanced cancer: a qualitative study
Source: BMJ Open. 2026 Jan 6;16(1):e102830. doi: 10.1136/bmjopen-2025-102830 (PMC12778277; doi:10.1136/bmjopen-2025-102830)
Supplement: online supplemental file 1 [file bmjopen-16-1-s001.docx]

# Interview guide – doctor’s experience of pain assessment with the Numeric Rating Scale (NRS)

Start by showing the Numeric Rating Scale (NRS). The following interview questions are opening questions. Ask follow-up questions where the interviewees can develop their thoughts and where there are ambiguities.

1. Do you use NRS at work?

2. Can you describe a typical situation when you use NRS at work and how you use NRS.

3. How do you ask a patient to use the NRS?

4. What do you think works well regarding NRS?

5. What do you think works poorly or less well regarding NRS?

6. What do you think of NRS?

7. Are there patients who cannot use the NRS? If so, how do you handle that?

8. How common is it that patients cannot use the NRS? Which patients cannot use NRS?

9. Does it happen that you don't think the answer seems right? If so, how do you think about it?

10. Is something about the NRS confusing or hard to understand?

11. How is the patients experiencing using of the NRS in your experience? (question added after interview number three)

12. What other pain assessment instruments do you use?

13. Do you have other views on the NRS?

14. Do you want to add something?

15. May the researchers contact you during the analysis process with any questions?
