# Supplementary material for: Palliative medicine physicians’ experiences using the Numeric Rating Scale for pain assessment in patients with advanced cancer: a qualitative study
Source: BMJ Open. 2026 Jan 6;16(1):e102830. doi: 10.1136/bmjopen-2025-102830 (PMC12778277; doi:10.1136/bmjopen-2025-102830)
Supplement: Supplementary file 2 [file bmjopen-2025-102830.reviewer_comments.pdf]

PEER REVIEW HISTORY

BMJ Open publishes all reviews undertaken for accepted manuscripts. Reviewers are asked to complete a checklist review form and are provided with free text boxes to elaborate on their assessment. These free text comments are reproduced below.

ARTICLE DETAILS

Title (Provisional)

Palliative medicine physicians' experiences using the Numeric Rating Scale for pain assessment in patients with advanced cancer – a qualitative study

Authors

Martinsson, Lisa; Brännström, Margareta; Fransson, Per; Andersson, Sofia

---

VERSION 1 - REVIEW

**Reviewer**	**1**
**Name**	**Yagasaki, Kaori**
**Affiliation**	**Keio University, Faculty of Nursing and Medical Care**
**Date**	**19-May-2025**
**COI**	**None**

Thank you for giving me the opportunity to peer-review your manuscript.

I understand that this study aims to explain the experiences of palliative care doctors using the numeric rating scale (NRS), which is commonly used for pain assessment.

Although the study is unique in that it targeted physicians who used the NRS rather than patients, it is difficult to understand the significance of conducting this study among physicians, based on the results and important issues presented.

Specific comments:

Strengths and limitations of this study: I believe that the use of reflexive thematic analysis in this case does not strengthen the research.

Introduction

I do not understand how the study aims to explain palliative medicine doctors' experiences of using the NRS for pain assessment.

It is difficult to understand the significance of this study from the Introduction.

NRS is a pain assessment tool. What is the significance of focusing on NRS and clarifying the experiences of physicians who use it?

Data analysis

Who analyzed the dataset and how was it analyzed?

How was the rigor of this study confirmed?

Results, Discussion, and Conclusion

Pain assessment in cancer-specific palliative care.

I believe it remains unclear whether the participants could have had similar experiences through the use of other scales, tools, or physical assessments, rather than the NRS. I am thus concerned about the accuracy of the results.

**Reviewer**	**2**
**Name**	**Chapman, Emma**
**Affiliation**	**University of Leeds**
**Date**	**12-Sep-2025**
**COI**	**None**

Thank you for the opportunity to review this manuscript.

This qualitative study describes the experience of experience of using the commonly applied NRS measure of pain from a healthcare professional perspective. Methods seem to be generally robust and the paper is clearly written. A suitable checklist is completed.

Please find below some specific points for your attention:

Page 4. The paper says that ‘No qualitative description of patients 'experience of using NRS, nor any study describing healthcare professionals' experience of using NRS as a pain assessment tool for patients with cancer has been found in the literature.’

I would like to see in the introduction some further rationale as to why addressing this research gap is necessary. A lack of existing data alone is not sufficient justification. What was the intended value of carrying out this research?

Page 5 – Can you clarify where participants worked. Is specialised palliative care in the hospital, hospice or community?

Page 6 – Can you comment on why reflective thematic analysis was chosen as your method? Reference 11 is on reflexive thematic analysis and this is the usual terminology.

Page 13. The study reports three Conclusions

1. The study shows the importance of working in a structured and individualized way when it comes to pain assessment in cancer in specialized palliative care.

2. Interpreting the tools for patients who are seriously ill is challenging for the team, but nonetheless, they are important for pain communication.

3. Future research needs to focus on developing pain assessment methods for patients who cannot self-report pain.

These to me seem to be general comments on pain assessment in palliative care rather than specifically relating to any new understanding of the experience of using the NRS resulting from this study.

What are the key conclusions that can be drawn from the data presented?

In addition, are there specific recommendations for practice, education or future research that can be made from the new understanding of the experience of using NRS, generated in this study?

**Reviewer**	**3**
**Name**	**Nagashima, Yasmin Guerreiro**
**Affiliation**	**Federal University of Rio Grande do Norte**
**Date**	**22-Sep-2025**
**COI**	**None**

Despite the study's apparent accuracy, the following limitations should be noted:

- The number of responders was low, which is unexpected given that digital interviews were used, as these methods can typically encourage participation.

- The insertion of a new question after approval by the ethics committee is a methodological issue that should be acknowledged as a limitation.

Considering all of this, the manuscript is well-written and contributes significantly to our understanding of pain evaluation in palliative care.

VERSION 1 - AUTHOR RESPONSE

Reviewer: 1
Dr. Kaori Yagasaki, Keio University
Comments to the Author:
Thank you for giving me the opportunity to peer-review your manuscript.
I understand that this study aims to explain the experiences of palliative care doctors using the numeric rating scale (NRS), which is commonly used for pain assessment.
Although the study is unique in that it targeted physicians who used the NRS rather than patients, it is difficult to understand the significance of conducting this study among physicians, based on the results and important issues presented.

Specific comments:
1. Strengths and limitations of this study: I believe that the use of reflexive thematic analysis in this

case does not strengthen the research.
Our answer: We have extended the methods section, including an explanation for the chosen method: A reflexive thematic analysis, following the six-phase approach outlined by Braun and Clarke, was conducted. This method was chosen for its flexibility and its emphasis on the researcher's active role in identifying and interpreting patterns of meaning across the dataset.
Introduction
2. I do not understand how the study aims to explain palliative medicine doctors' experiences of using the NRS for pain assessment.
It is difficult to understand the significance of this study from the Introduction.
NRS is a pain assessment tool. What is the significance of focusing on NRS and clarifying the experiences of physicians who use it?
Our answer: The choice of NRS was made because it is a common practice both in Sweden and internationally. Our research group has conducted a nationwide study about which pain assessment instruments are used in Sweden during the last week in life for patients with cancer within specialised palliative care, and found that the NRS is the most common, followed by the VAS [unpublished data]. This unpublished data will be presented by the author Lisa Martinsson at the Swedish national yearly conference for palliative care in Stockholm, 18-19 November 2025. We have added information about this to the introduction to clarify the rationale of choosing the specific tool.
Data analysis
3. Who analyzed the dataset and how was it analyzed?
Our answer: We have extended the methods section to clarify this.
4. How was the rigor of this study confirmed?
Our answer: We have addressed several points during this review process to ensure the study's rigour and clarify the method. We have extended the methods section and included examples of the coding process. We have also added more information about strengths and limitations to the manuscript.
Results, Discussion, and Conclusion
Pain assessment in cancer-specific palliative care.
5. I believe it remains unclear whether the participants could have had similar experiences through the use of other scales, tools, or physical assessments, rather than the NRS. I am thus concerned about the accuracy of the results.
Our answer: We have added more information in the introduction about the choice of the NRS as a focus for this study, please see our answer to point 2 above.
Reviewer: 2
Dr. Emma Chapman, University of Leeds
Comments to the Author:
Thank you for the opportunity to review this manuscript.
This qualitative study describes the experience of experience of using the commonly applied NRS measure of pain from a healthcare professional perspective. Methods seem to be generally robust and the paper is clearly written. A suitable checklist is completed.
Please find below some specific points for your attention:

Page 4. The paper says that 'No qualitative description of patients 'experience of using NRS, nor any study describing healthcare professionals' experience of using NRS as a pain assessment tool for patients with cancer has been found in the literature.'
I would like to see in the introduction some further rationale as to why addressing this research gap is necessary. A lack of existing data alone is not sufficient justification. What was the intended value of carrying out this research?
Our answer: The choice of NRS was made because it is a common practice both in Sweden and internationally. Our research group has conducted a nationwide study about which pain assessment instruments are used in Sweden during the last week in life for patients with cancer within specialised palliative care, and found that the NRS is the most common, followed by the VAS [unpublished data]. This unpublished data will be presented by the author Lisa Martinsson in the Swedish national conference for palliative care in Stockholm 18-19 November 2025. We have added information about this to the introduction to clarify the rational of choosing the specific tool.
Page 5 – Can you clarify where participants worked. Is specialised palliative care in the hospital, hospice or community?
Our answer: We have added information about this in Table 1.
Page 6 – Can you comment on why reflective thematic analysis was chosen as your method? Reference 11 is on reflexive thematic analysis and this is the usual terminology.
Our answer: Thank you for pointing this typo out. The correct description of our methods used is reflexive thematic analysis and we have corrected this in the manuscript.
Page 13. The study reports three Conclusions

1. The study shows the importance of working in a structured and individualized way when it comes to pain assessment in cancer in specialized palliative care.
2. Interpreting the tools for patients who are seriously ill is challenging for the team, but nonetheless, they are important for pain communication.
3. Future research needs to focus on developing pain assessment methods for patients who cannot self-report pain.
These to me seem to be general comments on pain assessment in palliative care rather than specifically relating to any new understanding of the experience of using the NRS resulting from this study.
What are the key conclusions that can be drawn from the data presented?
In addition, are there specific recommendations for practice, education or future research that can be made from the new understanding of the experience of using NRS, generated in this study?
Our answer: We have updated the conclusion.

Reviewer: 3
Dr. Yasmin Guerreiro Nagashima, Federal University of Rio Grande do Norte
Comments to the Author:
Despite the study's apparent accuracy, the following limitations should be noted:
1. The number of responders was low, which is unexpected given that digital interviews were used, as these methods can typically encourage participation.
Our answer: As described in the methods section, the research group considered the data material rich after conducting 14 interviews, and further data collection was thus not deemed necessary.
2. The insertion of a new question after approval by the ethics committee is a methodological issue that should be acknowledged as a limitation.
Our answer: There seems to be a misunderstanding. The semi-structured interview guide was slightly modified during the process, as was planned from the beginning and already mentioned in the ethics application. There was only one application to the ethics committee.
3. Considering all of this, the manuscript is well-written and contributes significantly to our understanding of pain evaluation in palliative care.
Our answer: Thank you

VERSION 2 - REVIEW

**Reviewer**	**2**
**Name**	**Chapman, Emma**
**Affiliation**	**University of Leeds**
**Date**	**27-Nov-2025**
**COI**	

Thank you for the opportunity to review this revision of the manuscript. I am satisfied that the changes made have addressed my previous comments and I am happy to recommend that the paper is now accepted for publication.

One final minor suggestion is to make it clearer whether the third point listed under strengths and limitations is viewed as a strength or limitation and why? The statement is 'The interviewed physicians in this study were all specialists, and almost all were specialists in palliative medicine'.

**Reviewer**	**3**

**Name**	**Nagashima, Yasmin Guerreiro**
**Affiliation**	**Federal University of Rio Grande do Norte**
**Date**	**21-Nov-2025**
**COI**	

The current version of the manuscript is clearly written, well-organized, and methodologically sound.
